# Construction and validation of a chemokine-related gene signature associated with prognosis, clinical significance, and immune microenvironment characteristics in cervical cancer

**DOI:** 10.1007/s12672-025-02973-7

**Published:** 2025-06-15

**Authors:** Tianjiao Huang, Renshuang Cao, Cong Gao, Jie Luo, Zhiyu Zhou, Kun Ma

**Affiliations:** 1 The First School of Clinical Medicine, Heilongjiang University of Chinese Medicine, Harbin, China; 2 Respiratory Department, Wangjing Hospital of China Academy of Chinese Medical Sciences, Beijing, China; 3 China Academy of Chinese Medical Sciences, Xiyuan Hospital, Beijing, China; 4 Graduate School, Tianjin University of Traditional Chinese Medicine, Tianjin, China

**Keywords:** Cervical cancer, Chemokine-related genes, Prognostic model, Immunotherapy

## Abstract

**Background:**

Cervical cancer (CC) remains a prevalent malignancy with significant mortality among women, highlighting the urgent need for reliable prognostic tools. While chemokines have emerged as pivotal regulators in tumor progression, their potential in constructing prognostic models for CC remains underexplored. This study aimed to develop a chemokine-related gene signature for outcome prediction and therapeutic guidance in CC patients.

**Methods:**

Transcriptomic data from The Cancer Genome Atlas (TCGA) cervical cancer cohort were analyzed to identify differentially expressed chemokine-related genes. Prognostic genes were screened through univariate Cox, multivariate Cox, and LASSO regression analyses, followed by the development of a risk stratification model. The model’s clinical relevance was evaluated by assessing its correlations with clinicopathological features, immune profiles, pathway enrichment, and therapeutic responses. A nomogram integrating risk scores and clinical parameters was constructed for survival prediction.

**Results:**

A nine-gene signature (CCL17, CXCL8, TNF, FOXP3, CXCL1, CCL20, ITGA5, CXCL3, CCR7) was established as an independent prognostic indicator. Kaplan–Meier analysis revealed significantly shorter overall survival (OS) and progression-free survival (PFS) in high-risk patients compared to low-risk counterparts (P < 0.05). Multivariate Cox regression confirmed the signature’s independence from conventional clinical variables (P < 0.05). The nomogram demonstrated robust predictive accuracy, with 1-, 3-, and 5-year survival AUC values of 0.805, 0.729, and 0.710, respectively. Distinct immune cell infiltration patterns were observed between risk groups, with low-risk patients exhibiting enhanced potential for immunotherapy and chemotherapy responsiveness.

**Conclusion:**

This study presents a clinically applicable prognostic model based on chemokine-related genes, providing insights for risk stratification and therapeutic decision-making in CC. Further validation through multicenter cohorts and mechanistic investigations of the identified genes are warranted to advance precision oncology strategies.

## Introduction

Cervical cancer (CC) persists as a predominant gynecological malignancy, presenting substantial global health challenges. According to 2022 GLOBOCAN statistics from the International Agency for Research on Cancer (IARC), CC ranks as the eighth most prevalent cancer worldwide, with 662,000 new cases (age-standardized incidence rate: 14.1/100,000), and the ninth leading cause of cancer-related mortality, accounting for 349,000 deaths annually (age-standardized mortality rate: 7.1/100,000) [1, 2]. Current understanding recognizes that tumorigenesis and progression of CC involve complex interactions between driver mutations and dynamic remodeling of the tumor immune microenvironment (TIME) [3]. While therapeutic advancements including platinum-based chemotherapy, molecularly targeted agents, and immune checkpoint inhibitors have improved clinical outcomes—particularly through combination regimens in metastatic settings—prognostic disparities persist [4]. Approximately 40% of patients present with advanced-stage disease due to frequent late-stage diagnosis driven by asymptomatic early progression and limited screening accessibility in resource-constrained regions. This clinical reality underscores the critical need for developing robust prognostic biomarkers to enable risk-adapted therapeutic strategies [5].

Immunotherapy has revolutionized oncology through its capacity to modulate antitumor immune responses, establishing new therapeutic paradigms across multiple malignancies [6]. Emerging evidence underscores the critical role of tumor microenvironment (TME) remodeling in determining immunotherapy efficacy [7], with chemokine networks emerging as pivotal regulators of immune cell recruitment and functional polarization [8]. In cervical cancer, while immune checkpoint inhibitors targeting PD-1/PD-L1 pathways have demonstrated clinical benefits, therapeutic resistance remains prevalent in 45–60% of patients. This limitation necessitates the identification of novel immunotherapeutic targets beyond the canonical PD-1/CTLA-4/PD-L1 axis [2, 9]. Chemokine-mediated crosstalk within the TME—particularly CXCL8-driven neutrophil infiltration and CCL20-CCR6-mediated regulatory T cell recruitment—has been mechanistically linked to immunosuppressive niche formation [10, 11]. Strategic targeting of these chemotactic axes may overcome current therapeutic bottlenecks by reprogramming the immune contexture [12].

Chemokines mediate signal transduction through G protein-coupled receptors (GPCRs), with their four structural subfamilies (CC/CXC/XC/CX3C) cooperatively regulating immune responses and tumorigenesis. In cervical cancer, these mediators remodel the tumor microenvironment (TME) by orchestrating angiogenesis, epithelial-mesenchymal transition (EMT) [13, 14], and metastatic dissemination, where CXCR7 has demonstrated prognostic significance through CXCL12-mediated migration regulation [15]. Although chemokine-targeted immunotherapies (e.g., checkpoint inhibitors) show clinical promise, the molecular regulatory networks underlying their context-dependent dual roles—balancing anti-tumor immunity with pro-metastatic signaling—remain poorly characterized in cervical carcinogenesis [16]. Our study systematically constructs a chemokine-oriented prognostic evaluation system through integrating bioinformatics analysis with a multi-omics validation framework, aiming to elucidate dynamic regulatory mechanisms of key signaling axes and provide theoretical foundation for developing precision therapeutic strategies based on chemokine microenvironment modulation [17, 18].

This study systematically analyzed the expression patterns of chemokine-associated genes in cervical cancer patients using public gene expression databases, with particular focus on their correlations with prognosis and clinical characteristics [19]. Through screening of pivotal chemokines, we established a prognostic risk prediction model for cervical cancer and evaluated its clinical utility [20]. Further investigations were conducted to explore the potential associations between chemokine expression profiles and tumor microenvironment (TME) composition as well as drug sensitivity [21]. Our findings demonstrate that this prognostic model holds substantial promise for clinical application in cervical cancer management.

## Materials and methods

### Identification of chemokine-related differentially expressed genes

Data Acquisition and Processing RNA sequencing transcriptomic data with matched clinicopathological information were obtained from 211 cervical squamous cell carcinoma (CESC) and cervical adenocarcinoma (CEAD) samples through The Cancer Genome Atlas (TCGA) database (https://portal.gdc.cancer.gov/). Intersection analysis between the TCGA dataset and 132 predefined chemokine-related genes identified 110 chemokine-associated genes in cervical cancer [22].

### Development of a chemokine-related gene signature for risk stratification in cervical cancer

Univariate Cox proportional hazards regression analysis was performed using the survival package in R (version 4.2.1) to identify prognostic biomarkers, with statistical significance defined as p < 0.05. To mitigate overfitting and optimize feature selection, LASSO-penalized Cox regression analysis was subsequently implemented via the glmnet package (version 4.1–4) through tenfold cross-validation. This approach enabled the identification of chemokine-related genes with the strongest prognostic associations for cervical cancer. The risk prediction model was formulated as:

$${\text{Risk Score}} = \Sigma \left( {\beta {\text{i }} \times {\text{Expi}}} \right)$$

where β represents the LASSO-derived regression coefficient and Exp denotes the normalized expression level of each selected gene. [23]

### Association between risk stratification and clinical characteristics in cervical cancer patients

To investigate the association between risk stratification and clinical characteristics in cervical cancer patients, we integrated risk scores with clinicopathological parameters using the limma R package (version 3.52.4). Univariate and multivariate Cox proportional hazards regression analyses were performed to assess the independent prognostic value of the risk score. Time-dependent receiver operating characteristic (ROC) curves were generated to evaluate the model’s predictive accuracy across different follow-up periods. Kaplan–Meier survival analysis with log-rank testing was subsequently conducted to compare survival outcomes among clinical subgroups [24].

### Integrating principal component analysis for risk prognostication modeling

Principal component analysis was conducted using the limma package to evaluate survival disparities between risk-stratified cohorts, with integrated visualization of TCGA patient survival status through scatter plots. This approach enabled systematic characterization of expression patterns encompassing nine pivotal genes across differential risk tiers within the prognostic model.

### Development and validation of a prognostic nomogram incorporating clinical parameters

Prognostic modeling was established through multivariable Cox regression analysis incorporating both the risk signature and clinicopathological parameters (gender, age, histological grade, FIGO stage) from the TCGA cervical cancer cohort. Nomogram construction was performed using the regplot package, with subsequent calibration curve generation via the rms and rmda packages to validate predictive accuracy. Subgroup stratification analysis revealed significant differential expression patterns of the Cervical Cancer Risk Score (CxRS-1) across TNM classifications, tumor grades, and age quartiles (P < 0.05, ANOVA with Tukey post-hoc). Time-dependent ROC analysis utilizing the timeROC package quantified the nomogram’s discriminative capacity at clinically relevant intervals (1-, 3-, 5-year OS).

### Functional annotation enrichment profiling

Using the clusterProfiler package in R, we conducted Gene Ontology (GO) analysis [25] and Kyoto Encyclopedia of Genes and Genomes (KEGG) pathway [26] enrichment analysis on chemokines in cervical cancer, unveiling a profound exploration of their functional significance [27]. We investigated the underlying biological pathways of differentially expressed genes in cervical cancer. Subsequently, we retrieved cancer hallmark-related gene sets from the Gene Set Enrichment Analysis (GSEA) [28–30] database and employed the “GSVA” package to calculate enrichment scores for these pathways. Additionally, we analyzed these pathways across different risk-stratified populations.

### Mutation analysis

Single nucleotide polymorphism (SNP) data for cervical cancer were extracted from the TCGA database to facilitate the investigation of the association between prognosis and cancer. The tumor mutational burden (TMB) for each patient was calculated using the “maftools” R package. TMB computation was restricted to nonsynonymous variants in coding regions, as these variants are more likely to have functional significance in tumor biology. Previous studies have demonstrated that using the median value is a common practice in TMB analysis, ensuring that our methodology aligns with established conventions in the field. Consequently, all patients were stratified into two groups based on the median TMB value: one group comprising samples with high TMB and the other with low TMB. When comparing high-risk and low-risk categories, we identified major variant genes with significant differences and explored the correlation between TMB and risk scores.

### Tumor microenvironment analysis

To determine whether there are distinct correlations between immune modulators and risk scores, we collected expression data of model genes and established immune modulators from the TCGA cohort. Subsequently, various algorithms were employed to analyze tumor microenvironment scores. The “oncoPredict” application was utilized to predict the therapeutic drug sensitivity across different risk subtypes of cervical cancer.

### Drug sensitivity analysis

We utilized the R package oncoPredict, which incorporates an in-built Ridge regression model to predict drug responses. The drug sensitivity data were sourced from two primary databases: the Cancer Therapeutics Response Portal (CTRP) (https://portals.broadinstitute.org/ctrp) and the PRISM (Profiling Relative Inhibitor and Substrate Mobility) database (https://depmap.org/portal/prism). Additionally, data from the Genomics of Drug Sensitivity in Cancer (GDSC) database (https://www.cancerrxgene.org) were employed to calculate the half-maximal inhibitory concentration (IC50) values for commonly used chemotherapeutic agents. The differences in drug sensitivity between the high-risk and low-risk groups were assessed using the Wilcoxon rank-sum test to identify statistically significant variations.

### Statistical analysis

R version 4.1.3 was used for statistical analysis. Statistical significance was determined based on a p-value of less than 0.05 and a false discovery rate (FDR) q-value of less than 0.05.

## Results

### Analysis of chemokine-related genes and prediction of mutation signatures in cervical cancer

**Fig. 1:**
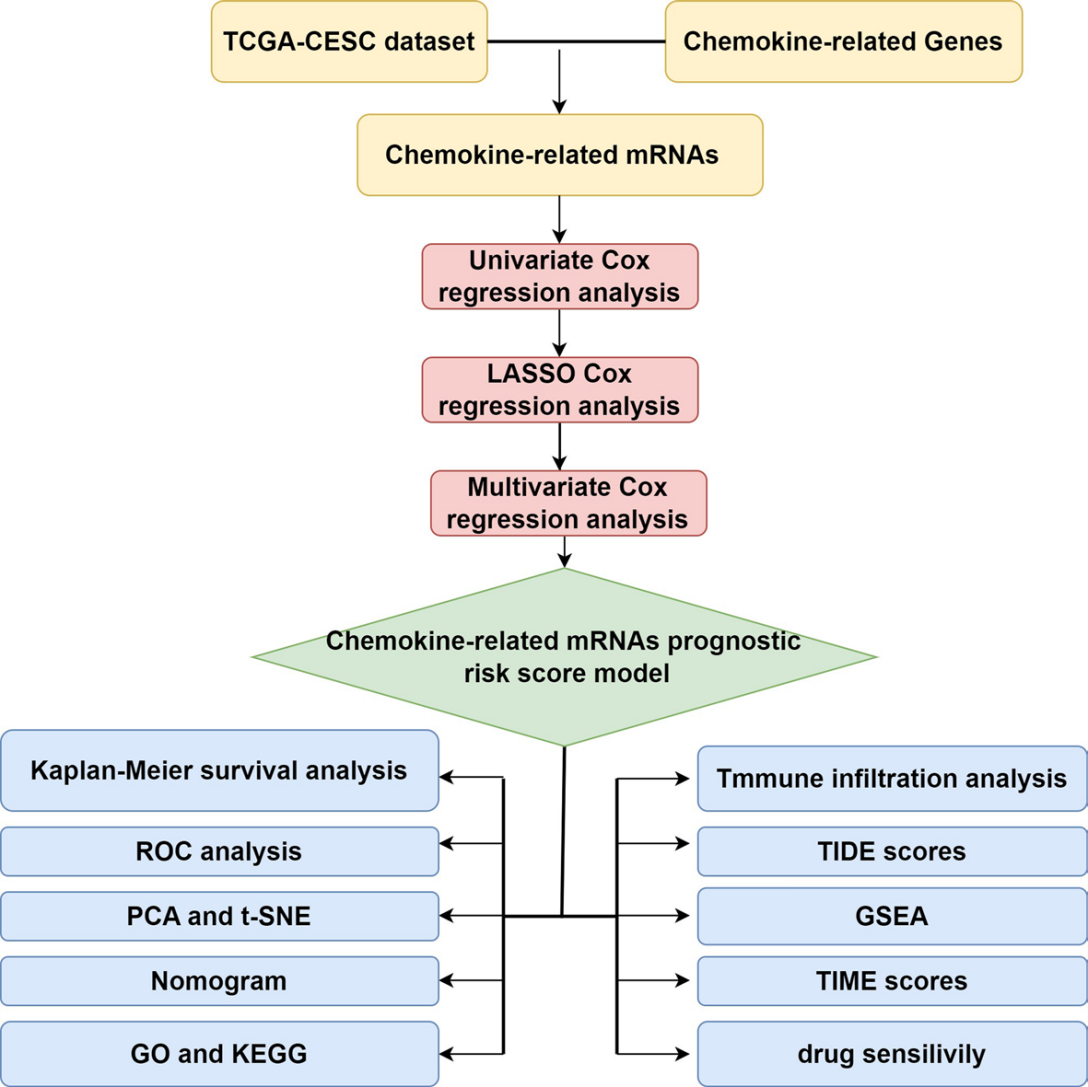
Flowchart of the study design

**Fig. 2:**
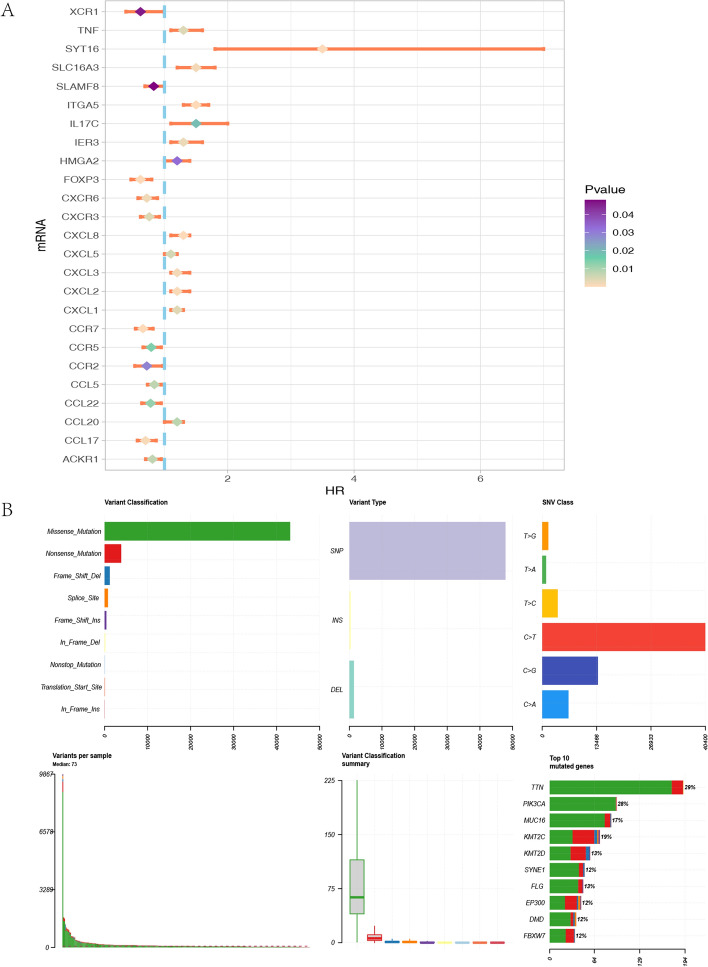
Screening of chemokines in cervical cancer (CC): **A** Prognostically significant chemokines identified through univariate Cox regression analysis (P < 0.05). **B** Genetic alteration landscape of chemokine-related genes in CC patients

The analytical workflow employed in this study is illustrated in (Fig. 1). The high- and low-risk groups were defined based on a risk scoring model constructed using prognostic chemokine-related genes. Specifically, RNA-seq data and clinical information for cervical cancer (CESC) samples were obtained from The Cancer Genome Atlas (TCGA) database (https://portal.gdc.cancer.gov/). A comprehensive collection of 132 chemokine-related genes was identified from published literature [31–33]. The intersection of all genes in 211 samples in TCGA with 132 chemokines associated with cervical cancer collected in the literature yielded 110 common genes [32]. This targeted approach focusing on biologically relevant chemokine pathways allowed us to identify the most functionally significant genes associated with cervical cancer progression. Subsequently, the prognostic significance of these 110 genes was assessed using univariate Cox regression analysis. As shown in (Fig. 2A), 25 genes demonstrated significant prognostic value, including 12 classified as low-risk and the remaining 13 as high-risk (p < 0.05). Furthermore, we evaluated genetic alterations in cervical cancer patients from the TCGA cohort. Among point mutations, missense mutations were the most prevalent type. Single nucleotide polymorphisms (SNPs) were identified as the predominant form of mutation in this population, with C > T transitions being the most frequent base substitution. Somatic mutation analysis revealed that mutation frequencies ranged from 8 to 29%, with TTN exhibiting the highest frequency at 29% (Fig. 2B).

### Construction of a prognostic risk model for patients with cervical cancer

**Fig. 3:**
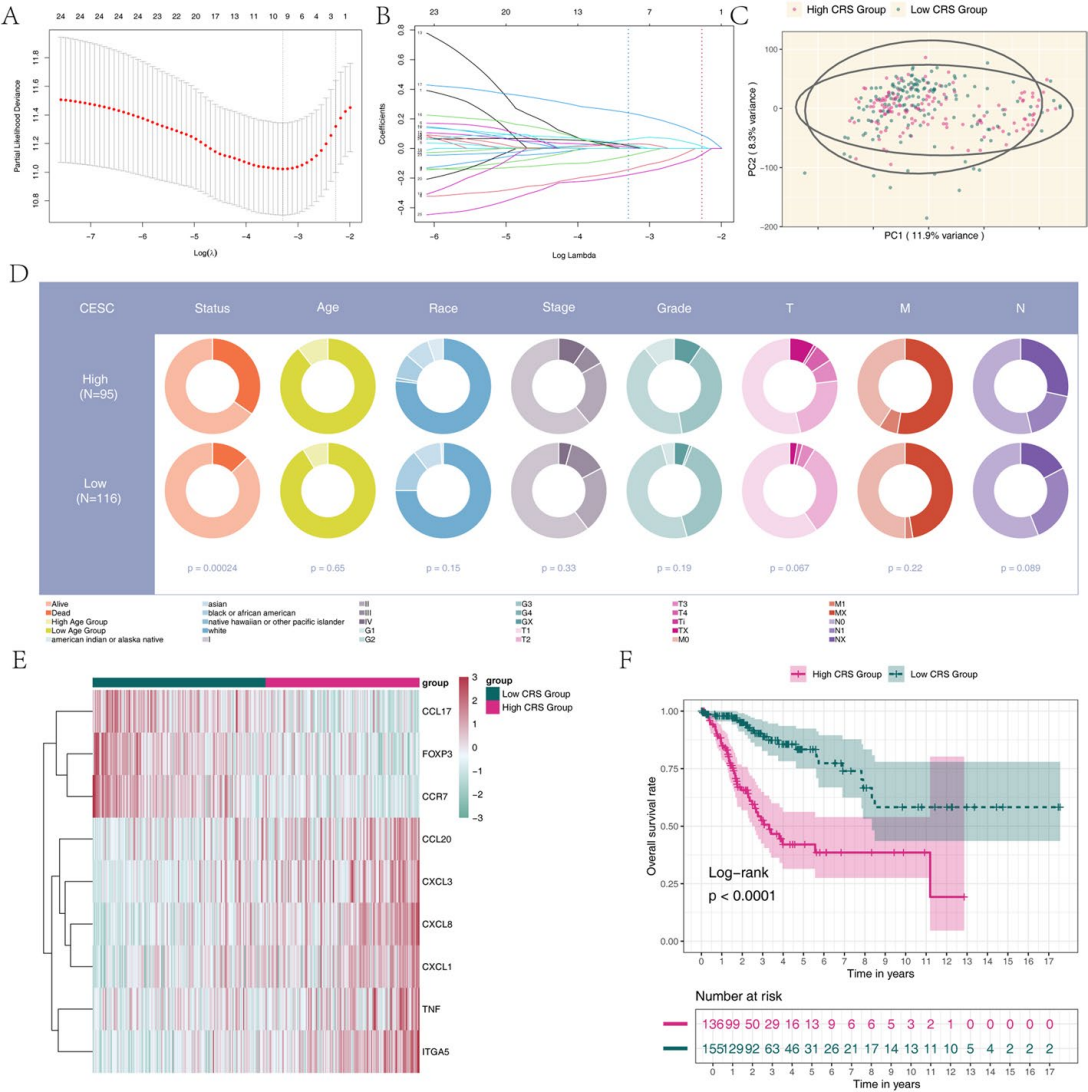
Development of a chemokine-related predictive model to assess the prognosis of cervical cancer patients: **A**, **B** Identification of chemokine-related prognostic signatures through LASSO regression analysis. The dashed line indicates the optimal value of λ. **C** Principal component analysis (PCA) of cervical cancer tissues and normal tissues. Low expression is represented in green, while high expression is indicated in red. **D** Stratification of risk groups based on the CRS threshold. **E** A heatmap illustrating the correlation between the expression levels of 9 chemokines and clinicopathological features in high-risk and low-risk cohorts. **F** The TCGA cohort demonstrated significant differences in overall survival (OS) between the low-risk and high-risk groups, as evidenced by Kaplan–Meier analysis

LASSO regression analysis was performed on the 25 prognostically significant chemokine-related genes to further refine our predictive model and mitigate potential overfitting. Through this rigorous statistical approach, 9 optimal chemokine-related genes were identified from the pool of 25 prognostic genes previously selected via univariate Cox regression analysis (Fig. 3A, B). Principal component analysis (PCA) was further utilized to distinguish cervical cancer samples (Fig. 3C, D), with patients identified as high-risk assigned to the red group and those classified as low-risk assigned to the green group. The formula provided below was used to calculate the risk score model: Risk Score = (− 0.160 * CCL17) + (− 0.005 * CXCL8) + (0.095 * TNF) + (− 0.257 * FOXP3) + (0.104 * CXCL1) + (0.059 * CCL20) + (0.330 * ITGA5) + (0.019 * CXCL3) + (− 0.279 * CCR7). Based on the observed threshold of the risk score in the TCGA cohort, patients were stratified into high-risk and low-risk groups. The expression levels of the nine model genes were visualized in a heatmap, and their associations with clinical variables were examined (Fig. 3E). Additionally, the Kaplan–Meier method was employed to assess potential prognostic differences in overall survival (OS) and progression-free survival (PFS) between the high-risk and low-risk groups. As shown in (Fig. 3F), patients in the low-risk group exhibited significantly better OS and PFS outcomes compared to those in the high-risk group (p < 0.001).

### Analysis of chemokine-related gene signatures in relation to prognosis and clinical characteristics

**Fig. 4:**
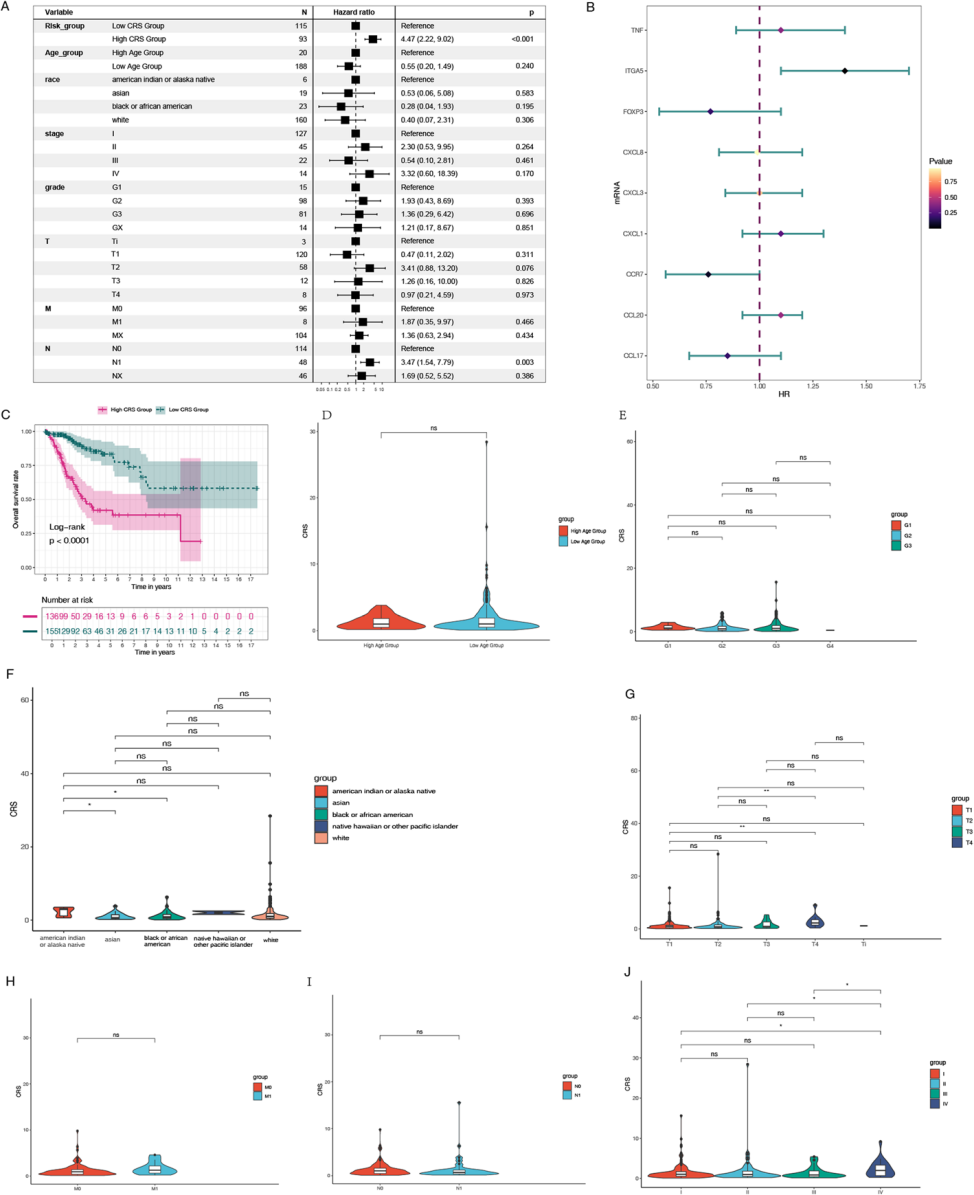
**A** Analysis of clinical characteristics based on risk scores. **B** Multivariate Cox regression analysis. **C** Kaplan–Meier analysis investigating the correlation between chemokine signatures and survival prognosis. **D**–**J** Subgroup analysis of clinical stage, NS indicates no statistical significance; *P < 0.05, **P < 0.01, ***P < 0.001

Multivariate Cox regression analysis was conducted using the TCGA dataset to evaluate the independent prognostic significance of chemokine-related gene signatures. In the multivariate Cox regression, N1 stage, high-risk score, and the genes TNF, ITGA5, CXCL1, and CCL20 were observed to be significantly associated with increased risk in cervical cancer patients (Fig. 4A, B). As shown in Fig. 4C, the area under the curve (AUC) values for 1-year, 3-year, and 5-year survival were recorded as 0.83, 0.79, and 0.78, respectively. Samples were stratified into specific subgroups based on age, ethnicity, clinical grade, and stage to explore the prognostic relevance of the signature in cervical cancer patients categorized by clinical variables. Our findings demonstrated that the Chemokine risk score (CRS) could effectively distinguish patients across different stages, such as T2 and T4 (Fig. 4D, J).

### Analysis of chemokine-related gene signatures

**Fig. 5:**
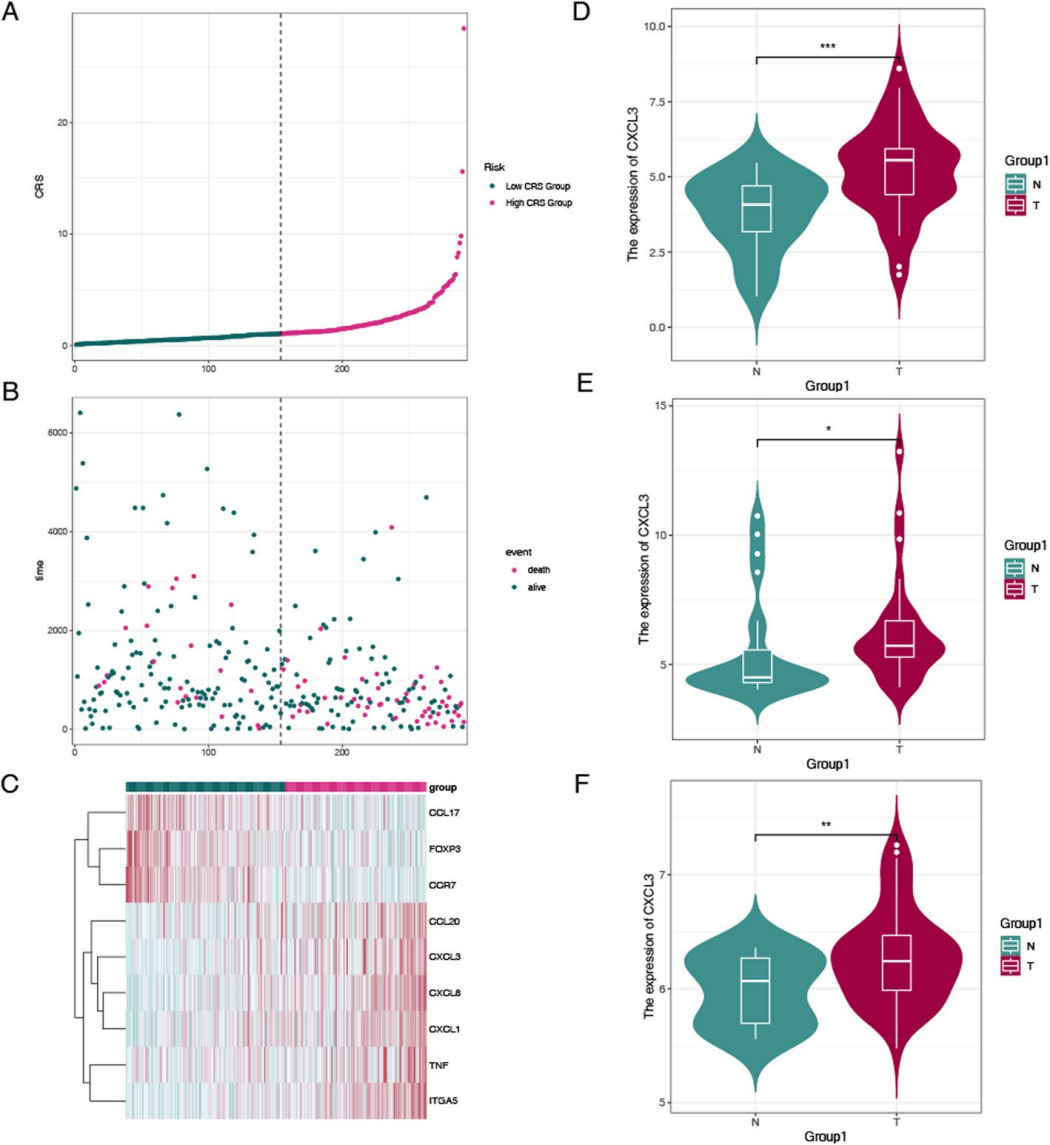
Construction of chemokine gene-related signatures. **A** Distribution of risk scores in the TCGA. **B** Survival status of patients in the TCGA. **C** Heatmap illustrating mRNA expression profiles in the TCGA dataset. **D**, **E**, **F** CXCL3 is also significantly highly expressed in tumor samples from external validation sets of GSE52930,GSE9750 and GSE3514

To better delineate survival differences between the high-risk and low-risk groups and to test the reproducibility of the risk prediction model, we stratified patients into two groups based on the chemokine risk model. The survival status of patients in the TCGA dataset was ranked and visualized using a scatter plot, which characterized the patients. Patient mortality gradually increased from the low-risk category to the high-risk category (Fig. 5A). The results were highly consistent with those observed in the TCGA training cohort (Fig. 5B). The heatmap results for the nine genes revealed elevated expression levels of high-risk chemokine-related genes, such as CCL20, CXCL3, CXCL8, CXCL1, TNF, and ITGA5, in the high-risk group. Conversely, the expression levels of low-risk chemokine-related genes, including CCL17, FOXP3, and CCR7, were reduced in the same progression (Fig. 5C). These findings demonstrate that the chemokine risk model performs well in stratifying cervical cancer patients into two distinct subtypes, high-risk and low-risk, based on their risk levels. In the meantime, we have conducted some internal validations using expression datasets, such as validating the core gene CXCL3. Expression analyses of CXCL3 showed that its expression was significantly higher in CC tumor tissues. These results were consistent with those obtained from the external validation datasets GSE52930,GSE9750 and GSE3514 (Fig. 5D–F).

### Development and validation of a nomogram survival model

**Fig. 6:**
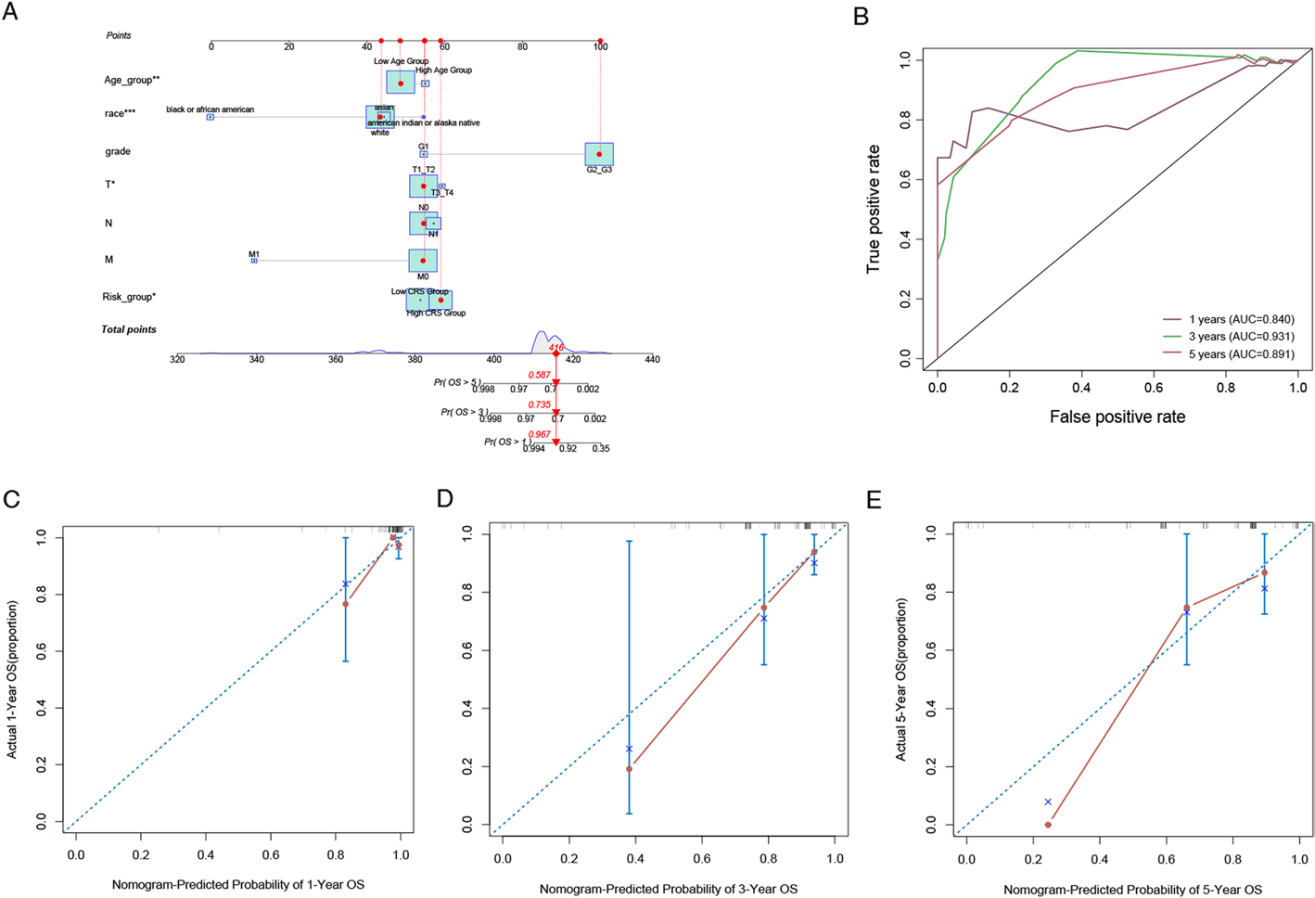
Development and evaluation of a chemokine-related nomogram survival model. **A** Nomogram constructed to predict 1-year, 3-year, and 5-year outcomes in cervical cancer patients. **B** ROC curve illustrating the prognostic value of the nomogram model. **C**, **E** Calibration curves estimating the survival rates of cervical cancer patients in the TCGA cohort at 1 year, 3 years, and 5 years

The comprehensive prognostic nomogram integrating our chemokine-based risk scoring system (CRS) with established clinicopathological parameters, including age stratification, ethnicity, tumor grade (G), TNM classification (T, N, M), and overall clinical stage, and (Fig. 6A). The nomogram assigned weighted points to each variable, with CRS demonstrating particular prognostic significance through its dichotomization into high- and low-risk categories. Optimal calibration was achieved for 3-year survival predictions (Fig. 6B). Furthermore, time-dependent ROC analysis validated the model’s discriminative capacity, yielding AUC values of 0.840, 0.931, and 0.891 for 1, 3, and 5-year survival predictions, respectively. In this scoring system, the cumulative total points enable direct estimation of 1-, 3-, and 5-year overall survival probabilities. For illustrative purposes, a representative patient with 341 total points demonstrated favorable survival probabilities of 96.7%, 73.5%, and 58.7% at these respective time points. Notably, CRS contributed the largest proportion of total points, underscoring its superior prognostic value compared to conventional clinical parameters. Model calibration was rigorously evaluated through calibration plots (Fig. 6C, E), where close alignment between predicted and observed outcomes (represented by the 45-degree reference line) indicated high predictive accuracy.

### Functional analysis of chemokine-related genes.

**Fig. 7:**
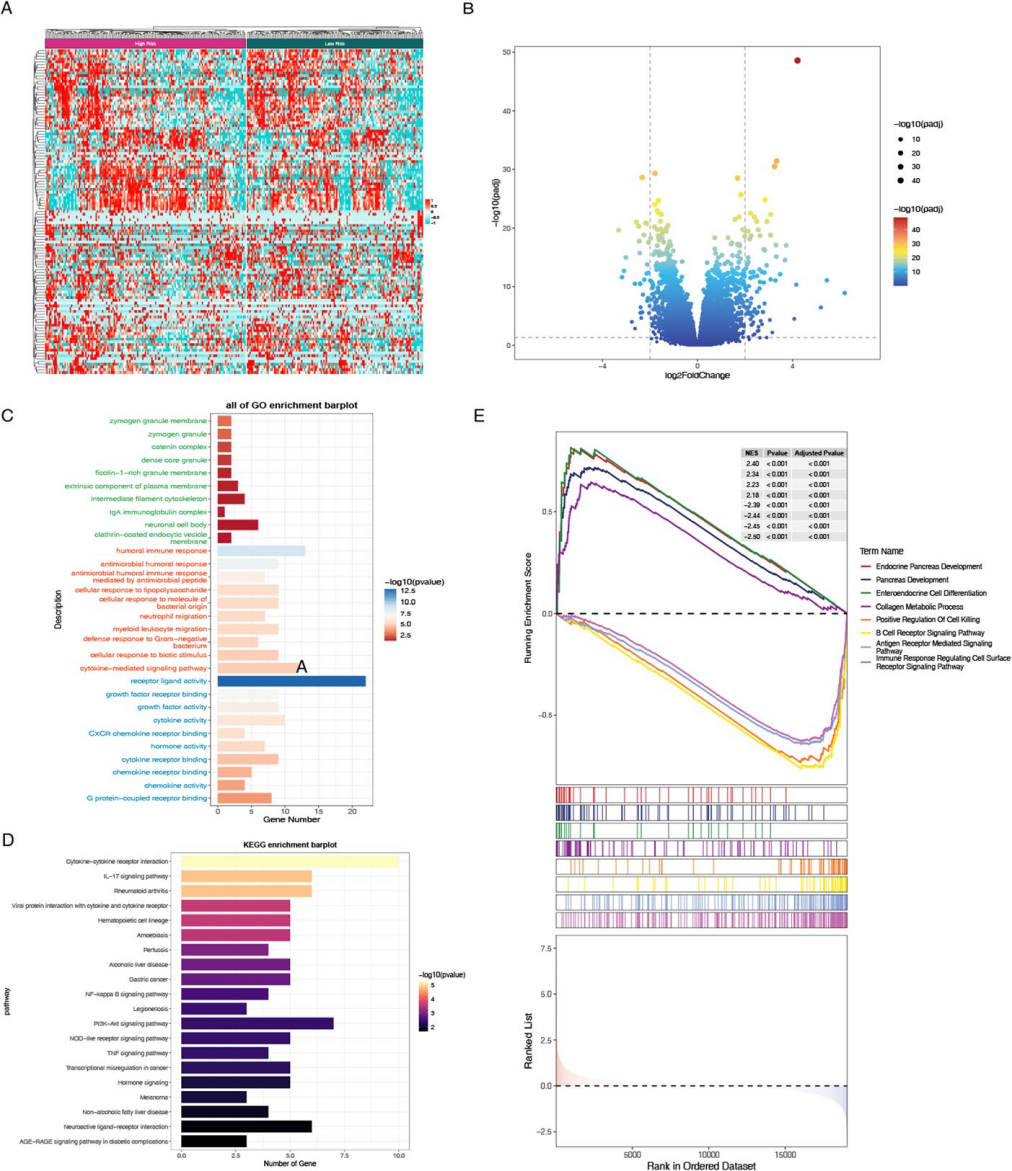
**A** Heatmap of differentially expressed chemokines between CC samples. **B** Volcano plot illustrating chemokine expression variability (|log2 fold change|> 1, adjusted P < 0.05). **C** GO pathway analysis results highlighting biological processes associated with the chemokine risk score. **D** KEGG pathway analysis results revealing enriched signaling pathways linked to the chemokine risk score. **E** Gene Set Enrichment Analysis (GSEA) of differentially expressed genes between the high-risk and low-risk groups

The difference analysis between the high-risk group and the low-risk group was carried out to obtain the difference genes, and the GSEA enrichment analysis between the two high and low risk groups was further discussed (Fig. 7A, B). Further enrichment analyses of the CRS identified in cervical cancer were performed using GO, KEGG, and GSEA to investigate its biological functions and associated signaling pathways. The results revealed that Biological Process (BP) showed significant enrichment in innate immune responses and antimicrobial defense mechanisms, particularly through cytokine-mediated signaling pathways (p < 1e-12) and cellular responses to bacterial components such as lipopolysaccharides (p < 1e-10). Key defense processes against Gram-negative pathogens were identified, including neutrophil migration (p < 1e-7) and myeloid leukocyte chemotaxis, suggesting robust leukocyte recruitment mechanisms. Humoral immune regulation was characterized by antimicrobial peptide-mediated responses (p < 1e-5), potentially implicating defensins or cathelicidins in pathogen clearance. Notably, multiple pathways converged on cellular responses to biotic stimuli, highlighting systemic adaptation to microbial challenges. Molecular Function (MF) demonstrated strong ligand-receptor interaction capabilities, particularly in chemokine receptor binding (p < 1e-10) and G protein-coupled receptor (GPCR) interactions (p < 1e-12). The significant enrichment of CXCR chemokine receptor binding (p < 1e-8) suggests specific involvement in neutrophil chemotaxis. Concurrently, cytokine and growth factor activities (p < 1e-7) were markedly elevated, indicating pleiotropic signaling functions. The dual presence of cytokine receptor binding and growth factor receptor binding implies cross-talk between immune activation and tissue repair mechanisms. Cellular Component (CC) localized to specialized immune effector domains, including ficolin-1-rich granule membranes (p < 1e-6) and zymogen granules (p < 1e-5), suggesting regulated secretion of antimicrobial factors. The prominence of clathrin-coated endocytic vesicles (p < 1e-7) implies active receptor internalization, potentially modulating chemokine signaling. Membrane-associated complexes were particularly enriched, with IgA immunoglobulin complexes (p < 1e-6) and extrinsic plasma membrane components (Fig. 7C). KEGG enrichment analysis primarily highlighted pathways such as cytokine-cytokine receptor interaction, transcriptional misregulation, neuroactive ligand-receptor interaction, and chemokine signaling pathway (Fig. 7D). Gene Set Enrichment Analysis (GSEA) revealed pronounced bidirectional dysregulation of critical pathways (adjusted P < 0.001). Upregulated pathways demonstrated robust activation of developmental programs, particularly endocrine pancreas development (NES = 2.40) and enteroendocrine cell differentiation (NES = 2.18), suggesting enhanced pancreatic islet maturation and hormone-secreting cell specification. Conversely, immune regulatory pathways exhibited significant suppression, including B cell receptor signaling (NES = − 2.50) and antigen receptor-mediated signaling (NES = − 2.45), indicative of impaired adaptive immune activation. Notably, the concurrent downregulation of collagen metabolic processes (NES = − 2.44) and positive regulation of cell killing (NES = − 2.39) implies compromised extracellular matrix remodeling and cytotoxic immune effector functions. This dichotomous signature – developmental activation coupled with immune suppression – may reflect a pathophysiological state balancing tissue regeneration against inflammatory damage, potentially relevant to autoimmune pancreatic disorders or immune-excluded malignancies (Fig. 7E).

### Analysis of the immune microenvironment and tumor mutation burden in the chemokine-related gene risk model

**Fig. 8:**
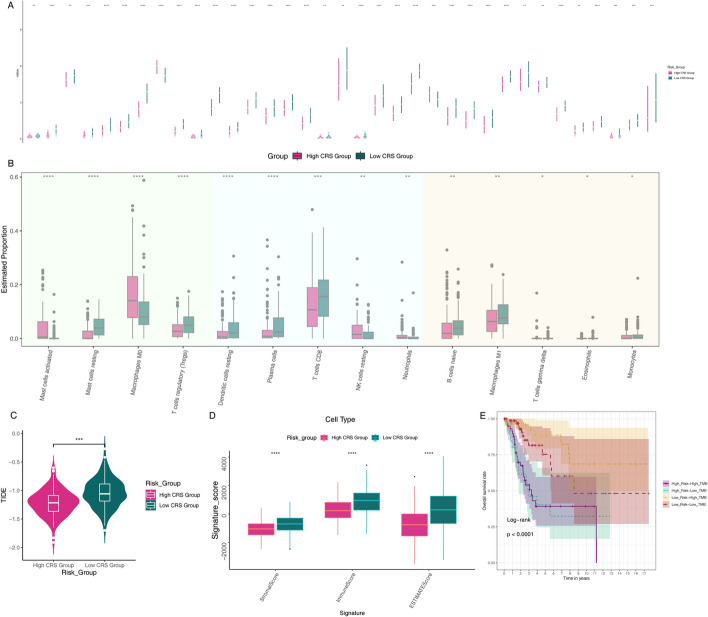
Analysis of immune cell infiltration in cervical cancer patients based on chemokine-related signatures **A** Relationship between cervical cancer risk subtypes and immune checkpoints. **B** Analysis of immune cell infiltration across different cervical cancer risk subtypes. **C** Tumor Immune Dysfunction and Exclusion (TIDE) scores across distinct risk subgroups. **D** Comprehensive scores for stromal and immune components across different risk subtypes. **E** Survival analysis of risk subgroups stratified by tumor mutation burden

Using estimation algorithms, we investigated the association between the chemokine risk model and anti-tumor immunity in cervical cancer patients. We observed a significant increase in the expression of multiple immune checkpoints across different risk subtypes of cervical cancer, including CD276 and NRP1 (Fig. 8A). In particular, expression levels of key immune checkpoints such as PDCD1 (PD-1), CTLA4, LAG3 and TIGIT increased significantly in high-risk groups, suggesting that high-risk patients may be more sensitive to immune checkpoint inhibitor treatment. Differences in immune cell expression levels were identified among distinct CRS risk subgroups, and immune cell infiltration in cervical cancer patients was calculated using the CIBERSORT algorithm based on the TCGA database. Notably, the H-CRS group exhibited higher expression levels of Macrophages M0 and NK cells (Fig. 8B).The infiltration of activated mast cells, T cells regulatory and CD8 + T cells in the tumor microenvironment of patients in the high-risk group increased significantly, indicating that these patients have a more active immune microenvironment, which may affect the efficacy of immunotherapy. It is worth noting that an increase in regulatory T cells is often associated with immunosuppression [34, 35], which may explain the poor prognosis of patients in the high-risk group. In the Tumor Immune Dysfunction and Exclusion (TIDE) analysis, the lower TIDE score in the H-CRS group was associated with better responses to immune checkpoint inhibition therapy (Fig. 8C), The results showed that the TIDE score in the high-risk group was significantly lower than that in the low-risk group, indicating that patients in the high-risk group may be more sensitive to immune checkpoint inhibitor treatment. Furthermore, to elucidate the differences in infiltrating immune cells among different risk groups, we compared them based on the ESTIMATEScore (the sum of stromal and immune scores), StromalScore (stromal cells in tumor tissue), and ImmuneScore (immune cell infiltration in tumor tissue). The results in (Fig. 8D)visually demonstrate that the TME scores (ESTIMATEScore, ImmuneScore, and StromalScore) were elevated in low-risk group patients compared to those in the high-risk group (p < 0.05). In terms of tumor mutation burden (TMB), the H-CRS-H-TMB group had the shortest survival time, while the H-CRS-L-TMB group exhibited the lowest survival rate (Fig. 8E), This finding has important clinical implications because high TMB is often considered a predictor of good response to immunotherapy, and our risk scoring model is able to further segment this group of patients and identify a patient population with a poor prognosis even under high TMB conditions.

### Drug sensitivity analysis in the chemokine-related gene risk model

**Fig. 9:**
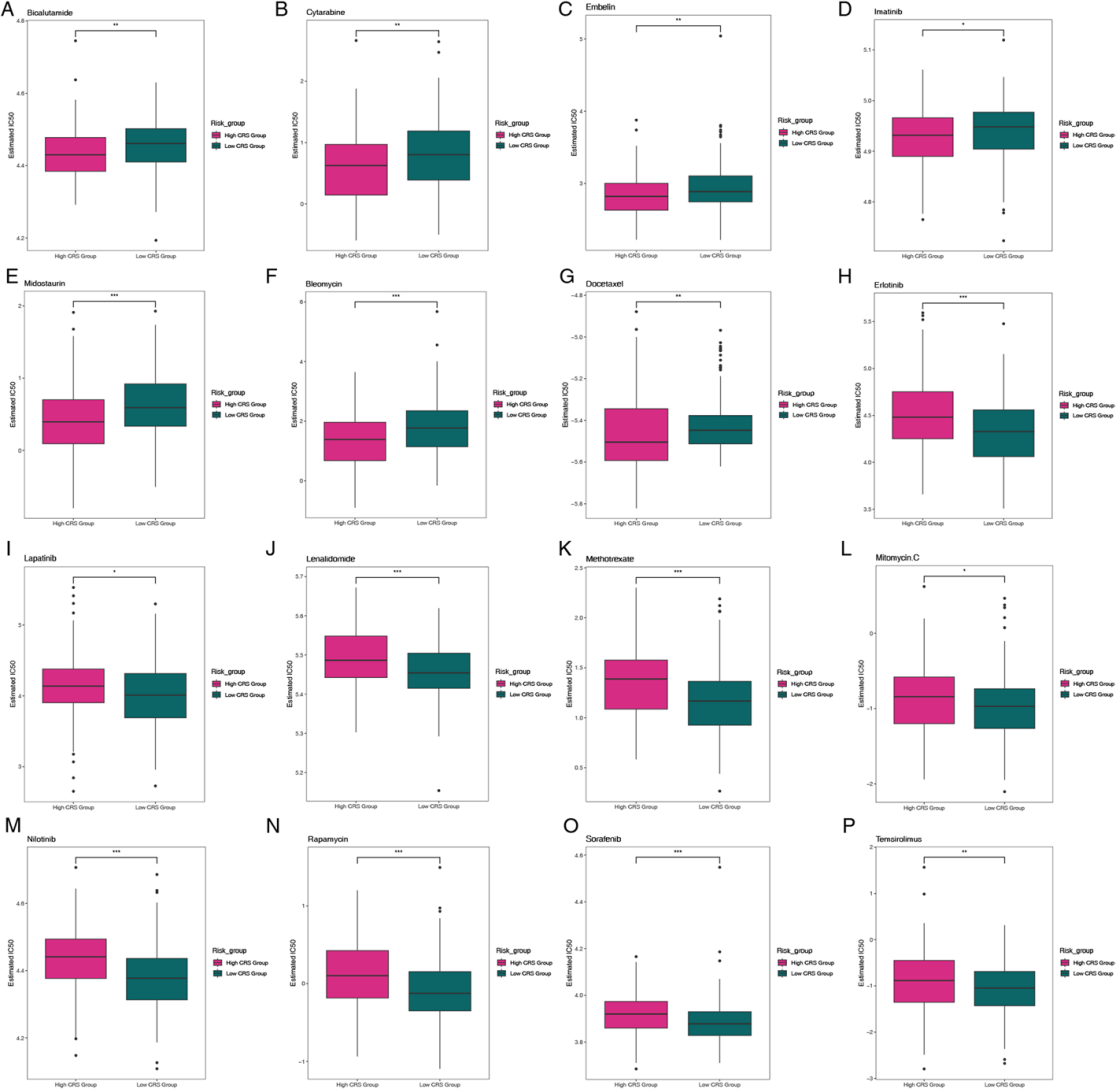
Correlation analysis between IC50 values of 16 chemotherapeutic agents and the chemokine risk score **A** Bicalutamide, **B** Cytarabine, **C** Embelin, **D** Imatinib, **E** Midostaurin, **F** Bleomycin, **G** Docetaxel, **H** Erlotinib, **I** Lapatinib, **J** Lenalidomide, **K** Methotrexate, **L** Mitomycin, **M** Nilotinib, **N** Rapamycin, **O** Sorafenib, **P** Temsirolimus

To further elucidate the differences in drug sensitivity among distinct risk subgroups of cervical cancer patients, we conducted a comprehensive analysis of the IC50 levels of various chemotherapeutic agents. The results revealed significant disparities in the IC50 values for 16 representative drugs, highlighting the potential for personalized treatment strategies based on chemokine-related gene expression profiles. Specifically, the low chemokine-related score (CRS) group exhibited elevated IC50 values for Bicalutamide, Cytarabine, Embelin, Imatinib, and Midostaurin, indicating reduced sensitivity to these agents [36]. The analysis demonstrated significant differences in IC50 values for 16 representative drugs (Fig. 9). This finding suggests that patients in the low CRS group may require higher doses or alternative therapeutic approaches to achieve effective treatment outcomes. Conversely, the high CRS group displayed higher IC50 values for Bleomycin, Docetaxel, Erlotinib, Lapatinib, Lenalidomide, Methotrexate, Mitomycin.C, Nilotinib, Rapamycin, Sorafenib, and Temsirolimus, indicating diminished sensitivity to these drugs. These results imply that patients in the high CRS group may benefit from alternative therapies or combination treatments to overcome potential resistance mechanisms. The observed differences in IC50 values may be attributed to variations in the expression of target proteins, metabolic pathways, and DNA repair mechanisms among the distinct risk subgroups. For instance, the elevated IC50 values for Bicalutamide and Imatinib in the low CRS group could be related to differences in androgen receptor and tyrosine kinase signaling pathways, respectively. Similarly, the higher IC50 values for Bleomycin and Docetaxel in the high CRS group may reflect alterations in DNA repair and microtubule dynamics. These findings underscore the importance of considering chemokine-related gene expression profiles when designing treatment regimens for cervical cancer patients, as they may provide valuable insights into drug sensitivity and resistance mechanisms. Further research is warranted to explore the underlying molecular mechanisms driving these differences and to validate these findings in larger cohorts. Additionally, the integration of these results with clinical data, such as patient outcomes and adverse events, could enhance the development of personalized treatment strategies and improve therapeutic efficacy in cervical cancer patients.

## Discussion

Cervical cancer is one of the most prevalent malignant tumors. According to reports from the World Health Organization (WHO), it is now recognized as the fourth most commonly diagnosed cancer globally and the third leading cause of cancer-related mortality. The pathogenesis of cervical cancer is complex, involving alterations in multiple genes and signaling pathways [37]. Advances in the fields of molecular biology and genomics have significantly contributed to a more comprehensive understanding of cervical cancer development. It has been discovered that abnormal expression or mutations of numerous genes are intricately linked to the onset and progression of this cancer [38].

In this study, we employed analytical approaches to identify chemokine-related genes in cervical cancer samples and ultimately developed a prognostic risk model based on chemokines for cervical cancer patients [39]. The established risk scoring model effectively stratified cervical cancer patients into high-risk and low-risk groups. The model identified five high-risk genes (TNF, CXCL1, CCL20, ITGA5, CXCL3) and four low-risk genes (CCL17, CXCL8, FOXP3, CCR7). Patients in the high-risk category exhibited unfavorable survival outcomes. High-risk genes (TNF, CXCL1, CCL20, ITGA5, CXCL3) typically promote tumor progression and immune evasion by inducing the expression of chemokines or modulating cellular responses to chemokines. For instance, in cervical cancer, TNF activates the NF-κB signaling pathway, inducing the expression of chemokines such as CXCL1 and CXCL8, which promotes the infiltration of neutrophils and macrophages, thereby facilitating tumor progression and immune evasion [40]. CXCL1, a member of the CXC subfamily of chemokines, primarily attracts neutrophils and eosinophils. It is highly expressed in various cancers and is closely associated with tumor invasion, metastasis, and immune evasion. In cervical cancer, CXCL1 promotes tumor cell migration and invasion through its receptor CXCR2 while modulating the immune microenvironment to suppress anti-tumor immune responses [41]. CCL20, a CC subfamily chemokine, primarily attracts dendritic cells and T cells. It is highly expressed in multiple cancers and plays a critical role in regulating the tumor immune microenvironment [42]. In cervical cancer, CCL20 modulates immune cell infiltration through its receptor CCR6, promoting tumor progression. ITGA5, a member of the integrin family, is involved in cell adhesion and migration. In cervical cancer, ITGA5 influences tumor cell responses to CXCL12 by regulating CXCR4 expression, thereby promoting cell migration and invasion [43]. CXCL3 [44], another CXC subfamily chemokine, shares biological functions with CXCL1 and CXCL2, primarily attracting neutrophils. It is highly expressed in various cancers and is closely associated with the inflammatory tumor microenvironment and immune evasion [29, 45]. For example, a study by Leonardo Fernandez-Avila reported that CXCL3 expression was significantly higher in cervical cancer tissues compared to normal cervical tissues. This finding suggests that CXCL3 could serve as a potential biomarker for cervical cancer. Ya-Ling Qi demonstrated that CXCL3 overexpression was associated with the generation of HeLa cell tumor xenografts in athymic nude mice [46]. This suggests that targeting CXCL3 could potentially enhance the efficacy of cancer treatments.

In contrast, low-risk genes (CCL17, CXCL8, FOXP3, CCR7) inhibit tumor progression by modulating immune cell infiltration and activity. In cervical cancer, low expression of CCL17 is associated with favorable prognosis. CCL17 regulates immune cell infiltration through its receptor CCR4, suppressing tumor progression [47]. FOXP3, a transcription factor, primarily regulates the function of regulatory T cells (Tregs). Although FOXP3 is not a chemokine itself, it can influence chemokine expression and immune cell infiltration by modulating Treg activity. In cervical cancer, low expression of FOXP3 is associated with favorable prognosis [48], as it inhibits tumor progression by regulating Treg activity. CXCL8, a CXC subfamily chemokine, primarily attracts neutrophils and eosinophils. In cervical cancer, high expression of CXCL8 is associated with the inflammatory tumor microenvironment and immune evasion. Similarly, low expression of CCR7 is associated with favorable prognosis, as it inhibits tumor progression by regulating immune cell migration [32, 49].

## Study limitations

However, this study has several limitations. First, our research constructed a chemokine-based risk model using retrospective data from public databases, but independent clinical trials were not conducted to validate our findings. Second, our prognostic risk model lacks comprehensive clinical information, which somewhat limits its ability to fully assess the prognosis and treatment outcomes of cervical cancer patients. Third, the study utilized transcriptomic data, which are more complex to interpret, thereby restricting its clinical applicability. Finally, the absence of in vivo and in vitro experiments to confirm the functional roles and mechanisms of the nine hub chemokines in cervical cancer necessitates further investigation.

## Conclusion

The model demonstrated high accuracy in predicting the survival prognosis of cervical cancer patients, establishing its potential as an independent prognostic indicator [50]. Functional analysis revealed that chemokine-related differentially expressed genes were significantly enriched in key biological processes, including innate immune responses and antimicrobial defense mechanisms, underscoring the critical role of chemokines in cervical cancer progression. Furthermore, distinct risk groups exhibited significant differences in their responses to various chemotherapeutic agents, offering diverse possibilities for personalized treatment strategies in cervical cancer patients. This study provides novel insights into the impact of chemokines on cervical cancer development. By constructing a prognostic model based on chemokines in cervical cancer, it enables accurate prediction of patient survival outcomes and facilitates better evaluation of individualized therapies, including immunotherapy and chemotherapy. The findings highlight the indispensable clinical significance of chemokines and offer valuable perspectives for therapeutic and prognostic strategies in cervical cancer [51].

In conclusion, this study elucidates the molecular characteristics of chemokines and explores their implications in cervical cancer. The results are expected to generate further interest and encourage additional research aimed at improving the treatment and prognosis of cervical cancer, ultimately enhancing patient survival rates and quality of life.

## Data Availability

The data for this study come from the Cancer Genome Atlas database (https://portal.gdc.cancer.gov/). The datasets generate during and analysis during the current study are available from the corresponding author on reasonable request.
